# Pseudomyopia and Its Association With Anxiety

**DOI:** 10.7759/cureus.17411

**Published:** 2021-08-24

**Authors:** Khizer Khalid, Jaskamal Padda, Sindhu Pokhriyal, Gazala Hitawala, Mohammd Saad Khan, Prejin Upadhyay, Ayden Charlene Cooper, Gutteridge Jean-Charles

**Affiliations:** 1 Internal Medicine, JC Medical Center, Orlando, USA; 2 Internal Medicine, Advent Health & Orlando Health Hospital, Orlando, USA

**Keywords:** anxiety, pseudomyopia, double vision, accommodative spasm, ciliary spasm

## Abstract

Pseudomyopia is an inappropriately excessive accommodation of the eye due to overstimulation or ciliary spasm, which leads to a marked approximation of the far point. Common symptoms of pseudomyopia include eye strain or fatigue, and it is classified as organic or functional. The latter is due to eye strain and functional increase in the ciliary tonus. Pseudomyopia can vary from being a transient condition or continue to progress to myopia. Head trauma is the most common cause followed by psychiatric illnesses, neurologic diseases, and drug-induced causes. There is an association between psychological stress-inducing events and pseudomyopia as it affects the autonomic nervous system. The human body counteracts anxiety by activating the parasympathetic nervous system, causing ciliary muscle contraction. Underlying psychiatric diseases in pseudomyopia patients have been reported in the past in multiple studies. Generalized anxiety disorder is the most common psychiatric illness associated with pseudomyopia with a positive correlation between anxiety-somatization scores and accommodation amount of the eye. It is strongly advised that a psychiatric consultation should be included in the multidisciplinary evaluation of every case. If patients have coexisting anxiety disorders, a multidisciplinary approach using psychiatric consultations, work environment changes, ocular exercises, and cycloplegic drugs can be used. This review aims to shed light on the association of psychiatric disorders such as anxiety with pseudomyopia.

## Introduction and background

Pseudomyopia or accommodative spasm occurs because of excessive constriction of the ciliary muscle which clinically manifests as blurred vision, distorted image, photophobia, and ocular pain. Symptoms are variable and can be unilateral or bilateral and constant or episodic [1,2]. Physiologically, when the eye wants to focus on a distant target, the ciliary muscles contract optimally to focus a clear image on the fovea. This reflex is known to have three components known as accommodation, convergence, and miosis and is mediated by a complex network of the parasympathetic nervous system (PNS) via the Edinger Westphal nucleus [1,3]. This reflex can malfunction for many reasons such as head trauma or excessive stress, resulting in an image of a near object to be projected in front of the retina instead of on the retina, which causes a blurred or distorted image [1,4]. This condition is called pseudomyopia, and unlike myopia, it is not associated with a large eye axis or an innate excessive curvature of the cornea or lens [2,4].

Although pseudomyopia is more prevalent in children, it is likely to reduce with age, making it more common in younger children. The prevalence of pseudomyopia is reported as approximately 24% in six-year-olds and 18% in 13-year-olds and is likely to increase further given the amount of time spent by children and adults on electronic devices such as smartphones, tablets, and laptops [5,6]. Head trauma, psychiatric disorders, demyelinating neurological disorders as well as posterior fossa tumors have been implicated as the leading causes of pseudomyopia [7-10]. This review article focuses on the association of pseudomyopia with psychiatric disorders such as anxiety, further describing its pathophysiology, clinical presentation, and diagnostic and therapeutic approaches toward managing this condition.

## Review

Defining pseudomyopia

Pseudomyopia is defined as an inappropriately excessive accommodation caused by overstimulation or ciliary spasm, which leads to a marked approximation of the far point. It is a type of myopia, also known as “short sight.” Hence, the person can naturally see objects which are near more clearly than distant objects and are called “short-sighted” [2]. This is usually a biological adaptive reaction to prolonged close work and stress. Long stretches of ciliary spasms make it difficult for the lens to relax when trying to focus on a distant object. Consequently, patients often experience blurred vision and asthenopia, more commonly known as eye strain or fatigue [11].

**Figure 1:**
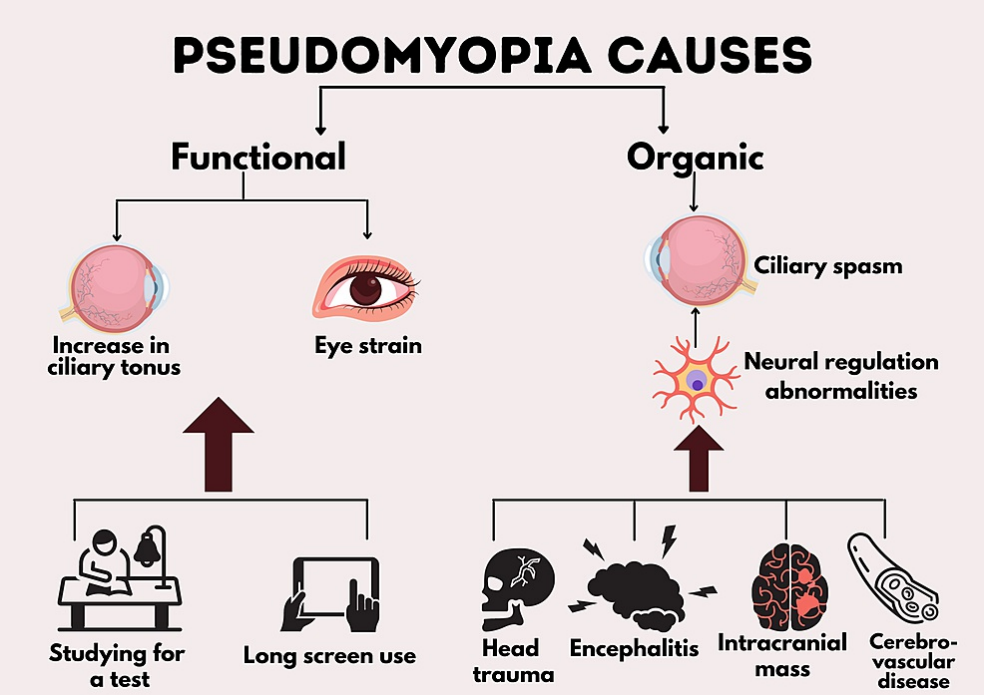
Causes of pseudomyopia. The figure shows the various factors that lead to pseudomyopia [12]. The image was created by the author (Dr. Gazala Hitawala).

The causes of pseudomyopia can be classified into two categories: functional and organic. Functional pseudomyopia is caused by eye strain and functional increase in the ciliary tonus. It occurs after a change in visual demands such as studying for a test or using a screen for long periods of time, commonly in teenagers and young adults who have active accommodation [12]. A true spasm of accommodation is considered pathological if excessive in duration and/or severity. Increases of 20 to 30 diopters have been documented due to persistent spasm in the ciliary muscle. Similar to functional pseudomyopia, it affects younger age groups (15 to 30 years), and the two may be considered at different points on the same spectrum [13]. Organic pseudomyopia can result from ciliary spasms due to neural regulation abnormalities as seen in head trauma, encephalitis, intracranial masses, and cerebrovascular diseases. The Edinger Westphal nucleus and the supranuclear controls of the third nerve nucleus, in particular, appear to be impacted [12]. Figure 1 summarizes the causes of pseudomyopia [12].

Pseudomyopia is a physiological adaptive response that is different from pathological myopia. The latter is commonly caused by abnormalities of axial length or less commonly by axial curvature. Myopia is commonly known as short-sightedness in which objects viewed at a distance appear blurred while near vision remains intact. It commonly results from abnormal elongation of the eyeball, which leads to the refractive image being generated in front of the retina [2].

Brain regions associated with pseudomyopia and anxiety

The effect of pseudomyopia on mental health is predominant because of the variability in the progression of the condition. It can vary from being a transient condition or can progress to myopia [14]. A 1979 study described “ocular hysteria” which commonly depicts “la belle indifference” and occurs as a result of emotional stress. The study further shed light on various diagnostic tests such as the psychogalvanometer which can help to identify psychological causes of visual disturbance [15]. In a 2021 case report, a 33-year-old female without any previous trauma reported a three-year history of compromised distant vision with unaffected near vision. Interestingly, the visual symptoms were preceded by her frequent migration abroad which induced tremendous stress. This psychological association was proven by the unstable refractive change seen with the large standard deviation of the manifest refraction during these periods of stress. The psychological stress stimulated a parasympathetic spasm which allowed the patient to control the pseudomyopia [3]. It is pertinent, that in response to stressors, the hypothalamus releases the corticotropin-releasing hormone that sends projections to the locus coeruleus of the brain stem. The locus coeruleus further sends direct signals to the sympathetic and parasympathetic nervous system [16]. With time, as the stressful situation fades, the PNS is activated to reduce the sympathetic response [17,18]. Thus, it can be concluded that physiologically the body counteracts the anxiety-inducing stressors by activating the PNS.

A case report of a 10-year-old girl demonstrated treatment refraction after multiple dosing of atropine. Her atropine dosage was eventually reduced to minimize the side effects. However, this resulted in a new-onset visual loss two days after the change in therapy. Notably, magnetic resonance imaging (MRI) of the orbit and brain was unremarkable. Consequently, considering a functional diagnosis, she was referred to a pediatric psychiatrist who diagnosed her with conversion disorder. She was diagnosed with functional visual loss associated with spasm of accommodation and was treated with psychotherapy and cycloplegic drugs [19]. From the above discussion, it is clear that psychological causes affect the autonomic nervous system. In addition, this case reinforces that the visual symptoms might not be associated with any abnormal brain imaging as the MRI for this patient was normal.

On the other hand, a 40-year-old black female presented with blurry distance vision after a motor vehicle accident. She also had a known medical history of depression and posttraumatic stress disorder. The brain imaging revealed the presence of a small cavernous in association with a developmental venous anomaly. The patient’s distance visual acuity for the right eye (OD) was 20/15 and for the left eye (OS) was 20/200. Her near visual acuity was OD 20/20 and for the OS was 20/20. She was diagnosed by neuro-ophthalmology with posttraumatic pseudomyopia. However, the cause for the pseudomyopia in this patient is unclear. It is postulated to be due to the disinhibition of the brain stem regulation of accommodation or the irritation of the parasympathetic third nerve nucleus [20].

In accordance with the above-discussed cases, it is established that there is an association between psychological stress-inducing events and pseudomyopia. Although there are various hypotheses to explain the correlation, there are fewer studies that acknowledge the involvement of the neuronal pathways or the specific brain regions which explain the occurrence of pseudomyopia in anxiety and vice-versa.

Double vision/vision’s association with anxiety

Previously, emotional distress was thought to be the most common precipitant of pseudomyopia [21]. However, recent studies show that head trauma is the most common etiology followed by psychiatric illnesses such as anxiety disorders and personality disorders. The other causes of pseudomyopia are reported to be neurologic diseases such as multiple sclerosis, posterior fossa anomalies, and pituitary tumors [6]. Drug-induced pseudomyopia due to amisulpride has also been reported [22].

**Figure 2:**
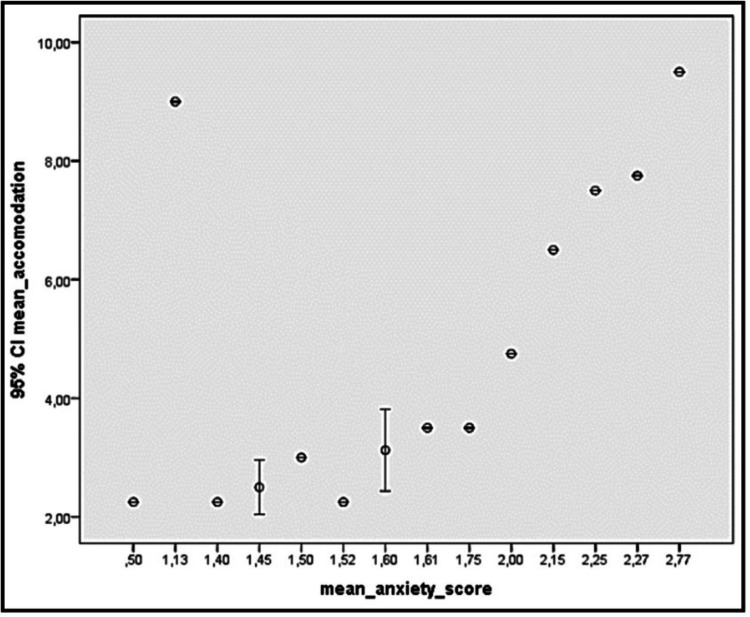
Correlation analysis between mean accommodation and mean anxiety score. The figure shows that the mean diopter measurement correlates with the mean anxiety score in the symptom checklist-90-R test. As the anxiety score increases, the measured myopia severity increases [23]. Copyright/License: Licensee Pak J Med Sci, Karachi, Pakistan. This figure is from an open-access article distributed under the terms and conditions of the Creative Commons Attribution License (http://creativecommons.org/licenses/by/3.0). No modifications were made to the original figure.

In a study done on 21 patients with pseudomyopia aged 12-18 years, 15 out of 21 (71.3%) participants were diagnosed and treated for a psychiatric diagnosis. Of them, five had generalized anxiety disorder, three had obsessive-compulsive disorder, three had panic disorders, one had social anxiety disorder, one had posttraumatic disorder, one had conversion disorder, and one had major depressive disorder. Interestingly, positive correlation (p = 0,010; r = 0,621 and p = 0,029; r = 0,546) was observed between anxiety-somatization scores and accommodation amount of eye (Figure 2) [23].

In another study done on movement disorders, convergence spasm was present in most of the psychogenic movement disorders cases (69%), with less likely occurrence on nonpsychogenic movement disorders cases and on controls. This study suggested that convergence spasms are associated with psychogenic movement disorders as well [24].

Underlying psychiatric diseases in pseudomyopia patients have been reported in multiple studies in the past [21,25-27]. In one case report of pseudomyopia, an underlying psychiatric cause was present in addition to an organic cause. This suggests that all patients diagnosed with pseudomyopia should be evaluated for psychiatric causes regardless of the etiology as it can coexist with organic causes, making it likely to be missed [28].

Many authors suggest that the presence of a psychiatric condition is the most likely cause of pseudomyopia if a discrete underlying etiology is not identified [29]. As the diagnosis of psychiatric causes with symptoms of pseudomyopia is challenging, this can increase patient burden because of invasive procedures such as carotid arteriography, pneumoencephalograms, and craniotomy, which was observed in five cases with an erroneous sixth nerve palsy diagnosis [30].

Diagnostic and treatment procedures of pseudomyopia associated with anxiety

When evaluating patients with myopia, especially those who have myopia associated with diplopia or strabismus, pseudomyopia or accommodative spasm must always be kept in mind as an alternative diagnosis [31]. Given the episodic nature of the disease, it can sometimes be challenging to establish a diagnosis on routine visual screenings [32]. It has been recommended to establish a diagnosis using dynamic retinoscopy during episodes of accommodative spasm. A significant difference in the degree of myopia detected on vision screening tests such as the photoscreener and cycloplegic refraction points toward the diagnosis of pseudomyopia [31,32]. The role of imaging in diagnosing pseudomyopia is limited and mainly pertains to cases afflicted with head trauma, although a raised intracranial pressure might have a more consistent association with pseudomyopia than positive MRI findings [33].

Fekete et al. in their study found that 71.4% of patients with pseudomyopia had some associated psychiatric disorders such as anxiety disorders, personality disorders, and depression. They also found a positive correlation between the severity of psychiatric disease and the severity of pseudomyopia [24]. Shetty et al. suggested that pseudomyopia was more often seen in women, and it is well known that anxiety disorders are more common in women than men [12,24]. In their study, Kara et al. strongly advised that psychiatric consultations should be a part of the multidisciplinary evaluation of every case of pseudomyopia [23].

Treatment modalities vary according to the underlying etiology of pseudomyopia and, more often than not, pseudomyopia does not resolve spontaneously. Therefore, therapeutic options have most often been focused on reducing the ciliary and thus the accommodative spasm. In patients who have pseudomyopia with coexisting anxiety disorders, a multidisciplinary approach has been used to manage and treat pseudomyopia which includes using psychiatric consultations and work environment changes, ocular exercises, and using cycloplegics to relax the ciliary muscle spasm. Although stronger cycloplegics like atropine show better results compared to weaker cycloplegics like homatropine, homide, and tropicamide, they are associated with systemic side effects and ocular discomfort. This required tapering of doses until these symptoms were manageable or required switching to weaker cycloplegics [3,6,7].

Hyndman summarized how over the years, the use of psychiatric medications in carefully selected cases has led to the resolution of pseudomyopia. He described the resolution of accommodative spasms with the use of sertraline, diazepam, valproate, promethazine, bromocriptine, and Amytal in different scenarios where pseudomyopia was associated with psychiatric disorders like anxiety [6]. Hussaindeen et al. discussed using vision therapy encompassing accommodative facility exercises with plus lenses and Hart chart even after having stopped the cycloplegic agent [7].

When plus glasses do not work, Prerana et al. suggest prescribing cycloplegics like atropine as the next course of action [34]. Shah et al., in their study on the efficacy of different cycloplegics in the treatment of accommodative spasm, found homide eye drops to be less efficacious compared to atropine [35]. Punctal pressure for a few seconds after instilling atropine can decrease systemic side effects by reducing systemic absorption [7]. Having said that, if a patient cannot tolerate atropine due to its side effects such as conjunctivitis, dermatitis, eye pain, or irritation, ophthalmologists often prefer using milder cycloplegics like homide [35,36]. Kaczmarek et al. have discussed the use of botulinum toxin in treating pseudomyopia and managing it like any other muscle dystonia [37]. Radical treatments that permanently paralyze a patient’s ability to accommodate for life include clear lens exchanges which are reserved for rare treatment-unresponsive cases [38].

## Conclusions

Pseudomyopia can be triggered by visual and psychological stress. These stresses are managed by the body with an increase in the PNS activity, which then causes spasms of the ciliary muscles. Psychological evaluation and diagnostic tests such as psychogalvanometer can help to identify psychological causes of visual disturbance. During evaluation for myopia, alternative diagnoses such as pseudomyopia or accommodative spasm must always be kept in mind. Functional pseudomyopia has shown some benefit from psychotherapy, ocular exercises, and/or cycloplegic drugs. Further studies are needed to confirm the hypotheses explaining the association between psychological stress and pseudomyopia, to identify neuronal pathways or the specific brain regions which explain the occurrence of pseudomyopia in anxiety, and to explore further treatment options and guidelines.
